# Activity-dependent plasticity of hippocampal place maps

**DOI:** 10.1038/ncomms11824

**Published:** 2016-06-10

**Authors:** Philipp Schoenenberger, Joseph O'Neill, Jozsef Csicsvari

**Affiliations:** 1 Institute of Science and Technology Austria, Am Campus 1, A–3400 Klosterneuburg, Austria

## Abstract

Hippocampal neurons encode a cognitive map of space. These maps are thought to be updated during learning and in response to changes in the environment through activity-dependent synaptic plasticity. Here we examine how changes in activity influence spatial coding in rats using halorhodopsin-mediated, spatially selective optogenetic silencing. Halorhoposin stimulation leads to light-induced suppression in many place cells and interneurons; some place cells increase their firing through disinhibition, whereas some show no effect. We find that place fields of the unaffected subpopulation remain stable. On the other hand, place fields of suppressed place cells were unstable, showing remapping across sessions before and after optogenetic inhibition. Disinhibited place cells had stable maps but sustained an elevated firing rate. These findings suggest that place representation in the hippocampus is constantly governed by activity-dependent processes, and that disinhibition may provide a mechanism for rate remapping.

## Abstract (web summary)

Place cells in hippocampus encode a map of space, however the role of activity in place map stability is not known. Schoenenberger and colleagues optogenetically manipulate hippocampal firing rates within place fields and show lasting changes in spatial firing patterns through two separate mechanisms.

## Graphical abstract

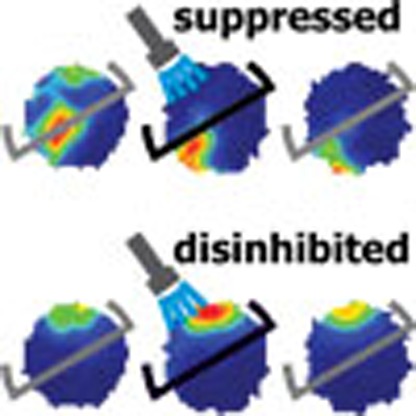

Hippocampal place cells fire in relation to place and together they form a cognitive map of the surrounding space^1^. Different environments are represented by an orthogonal arrangement of place fields, which are rapidly formed during exploration of novel environments. Once a new map is formed, it remains stable over many subsequent exposures to the same environment^2^. However, in certain conditions, place cells can remap even in familiar environments, for example, during goal-oriented spatial learning^3^,^4^. Furthermore, changing features of the recording enclosure can lead to rate remapping in which the in-field firing rates of cells change without affecting the location of their place fields^5^. Both rate and field remapping may occur as a result of changes in afferent inputs, as might be expected by the rapid emergence of spatially selective firing on exposure to a new environment^6^. However, both selecting the region of space in which a cell fires and the intensity of firing within its place field may be, in part, governed by activity-dependent processes and therefore by experience^7^,^8^.

From intracellular recordings we know that hippocampal place cells depolarize their membrane potentials when they fire inside their place fields^9^,^10^,^11^. Several place-selective subthreshold depolarization fields also exist^10^, suggesting that many different spatial inputs may compete initially to influence the firing of a place cell. Place cells receive both direct entorhinal cortical and intrahippocampal inputs. Direct entorhinal cortex inputs are sufficient for place cell activity, because CA1 cells can form place fields even when CA3 is lesioned^12^. Furthermore, input from the medial entorhinal cortex (MEC) is spatially modulated and spatially firing MEC cells including the grid cells and border cells send direct projections to the hippocampus^13^,^14^,^15^. Border cells, by marking environmental boundaries are thought to provide sensory information in relation to external spatial cues^16^, whereas grid cells may represent path-integration computations^17^,^18^,^19^,^20^. When grid cell activity is disrupted by the inactivation of the medial septum, the spatial selectivity of place cells remains, suggesting that the inputs from border cells and other spatially tuned MEC cells are sufficient for place-related firing in the hippocampus^21^. The CA3 region is thought to provide pattern completion, which may help spatial coding in familiar environments in partial-cued condition, for example, in large recording enclosures, when path-integration information from grid cells accumulates errors^22^,^23^,^24^,^25^. New place fields can be generated by inducing dendritic plateau potentials on CA1 dendrites through simultaneously activating CA3 and entorhinal inputs^26^.

However, it is not understood how such spatially selective inputs are selected once a cell forms a new place field. It is expected that plastic changes between those inputs and the CA1 neurons plays a role. Indeed, several lines of evidence suggest that plasticity constantly influences hippocampal spatial maps including their stability and expression. The stabilization of the place fields and their later reinstatement require NMDA (*N*-methyl-D-aspartate) receptors (NMDAR) and protein synthesis^27^,^28^. Moreover, the induction of long-term potentiation triggers the remapping of place cells^29^. During exploration, place fields are likely to be stabilized through activity-dependent processes, as sustained firing inside a cell's place field is required before a new place field is stabilized^7^,^30^. Furthermore, place field shape itself has been suggested to be modulated by activity-dependent plasticity; repeated traversal along a single path results in an expansion of the place field towards earlier portions of the track^31^,^32^. This asymmetric expansion may reflect spike timing-dependent plasticity rules^33^,^34^,^35^. Moreover, the development of such place field asymmetry is NMDAR dependent, further confirming the involvement of activity-dependent synaptic plasticity in this process^36^.

These findings suggest that the shape and stability of place fields can be regulated by the firing pattern of place cells inside their place field. To investigate how the activity of place cells might itself influence future place field expression, we used an optogenetic approach to manipulate the firing rates of hippocampal place cells. In doing so, we also tested the hypothesis that activity-dependent plastic changes constantly modulate the shape and stability hippocampal spatial firing patterns. We found that suppressing the firing rate of the place cells inside their place field can lead to the subsequent remapping of their place fields. By contrast, facilitating the firing rate of place cells through disinhibition triggered lasting increase of their firing rate inside their place fields. These results demonstrate that activity-dependent mechanisms can trigger lasting changes in the spatial firing of place cells.

## Results

### Optogenetic manipulation of CA1 activity

**Figure 1:**
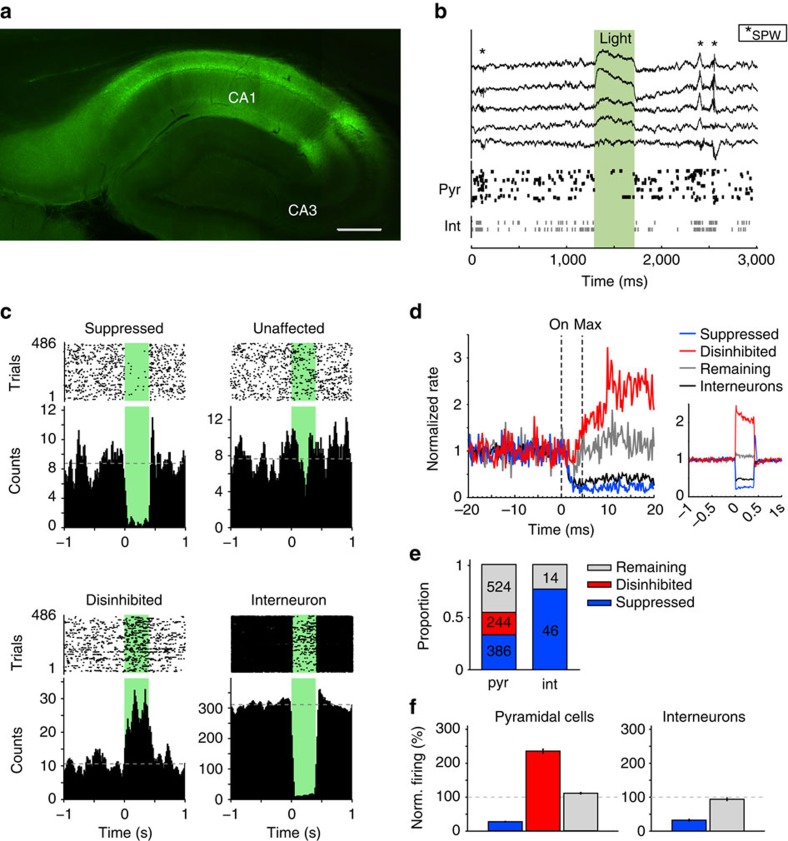
Optogenetic manipulation of single-unit activity in dorsal CA1 during sleep/rest. (**a**) NpHR-YFP expression in the dorsal CA1 area of a virus-injected rat. Sagittal section; scale bar, 400 μm. (**b**) Brain activity in dorsal CA1 during slow-wave sleep with NpHR activation. Top: wide-band signal from individual channels from five different tetrodes. Asterisks mark sharp wave/ripples (SPW). Bottom: Raster plot showing activity of pyramidal cells (Pyr) and interneurons (Int). Green area marks 400 ms laser pulse. Laser pulses were applied at 0.5 Hz frequency intervals. (**c**) Raster plots and peristimulus histograms showing light responses of representative suppressed (top left), unaffected (top right) and disinhibited (bottom left) pyramidal groups and a suppressed interneuron (bottom right). Dashed line: baseline firing activity. (**d**) Time course of light responses (left): firing rate 20 ms before and after onset of laser pulse. Dashed lines: on, laser onset; max, maximal inhibition of pyramidal cells and interneurons. Right: response to the entire 400-ms laser pulse. (**e**) Proportions of suppressed, disinhibited and remaining cells during rest. Numbers indicate cell counts. Total CA1 units, *n*=1,154 pyramidal cells, *n*=60 interneurons from 18 recording days in 5 animals. (**f**) Light responses for different cell categories relative to baseline. Error bars represent mean+s.e.m.

We expressed Halorhodopsin (NpHR-YFP) under control of the CaMKIIα promoter in the CA1 region of the hippocampus in (*n*=5) rats using an adeno-associated virus (AAV; Fig. 1a). A microdrive apparatus with 15 tetrodes and one 200-μm optic fibre centred in the middle of the tetrode electrode bundle was implanted to target the dorsal CA1 region of the hippocampus. This enabled us to record the activity of both pyramidal cells (*n*=1,154) and interneurons (*n*=60), while the animal explored different arenas or rested in a sleep box, in the presence or absence of laser stimulation. Light was applied in one of the exploration sessions (that is, light-on session) in a discrete segment of the environment (that is, light zone). We assessed the effect of firing rate disruption on the stability of previously established fields in familiar environments and the formation of new fields in novel environments using two different recording protocols, performed on different days (Supplementary Fig. 1, see below). Light application did not influence the speed of the animal, nor did it cause a bias in the visit probability of the light zone considering their mean values (Supplementary Fig. 2a) or their ratios (Supplementary Fig. 2b). At the end of each day's experiment, light pulses of 400 ms duration were applied during an immobility session, to classify the light responses of cells, independent of their place-related firing (Fig. 1b).

### Single-unit light responses

The activity of a third of pyramidal neurons (386/1,154, 33.4%) was strongly reduced during illumination (suppressed) in the rest box (see Methods for the criteria used for response classification). Surprisingly, 21.1% of the pyramidal neurons (244/1,154) increased their firing as a result of the light application (disinhibited; Fig. 1c,e and Supplementary Table 1). This increase in pyramidal cell activity may have occurred as a result of reduced interneuron activity. Indeed, the activity of 76.7% of the interneurons (46/60) was suppressed during the laser flashes (Fig. 1c,e). To investigate whether the reduced interneuron activity was the result of direct inhibition by NpHR or a network effect resulting from reduced pyramidal cell activity, we performed immunolabelling. In agreement with a previous report^37^ showing that the CaMKIIα promoter can drive expression in both pyramidal cells and interneurons with AAV-mediated gene delivery, we observed expression of NpHR in both somatostatin- and parvalbumin immunopositive interneurons (Supplementary Fig. 3). Moreover, suppressed pyramidal cells and interneurons responded 1–2 ms after the light onset, whereas pyramidal cells that increased their activity exhibited a >5-ms delay (Fig. 1d). Taken together, these data suggest that the firing increase observed in a subgroup of pyramidal cells was caused by disinhibition, due to the optical inhibition of NpHR-expressing interneurons. Suppressed pyramidal cells exhibited a 78% reduction, while 134% rate increase was seen for the disinhibited cells (Fig. 1f).

### Reorganization of CA1 place fields in a familiar environment

We examined how disruption of spatial firing patterns affected the stability of previously established place fields, using the ‘familiar' paradigm (see Supplementary Fig. 1a). Here we recorded an exploration session in a familiar environment with no light stimulation, which served as a reference point for place field and firing rate of each place cell. This was followed by an exposure to a novel environment and then back to the familiar environment, this time with laser stimulation in the light zone. A further exploration session was recorded in the familiar environment, to establish if light application had resulted in a sustained modification to either rate or place field expression.

**Figure 2:**
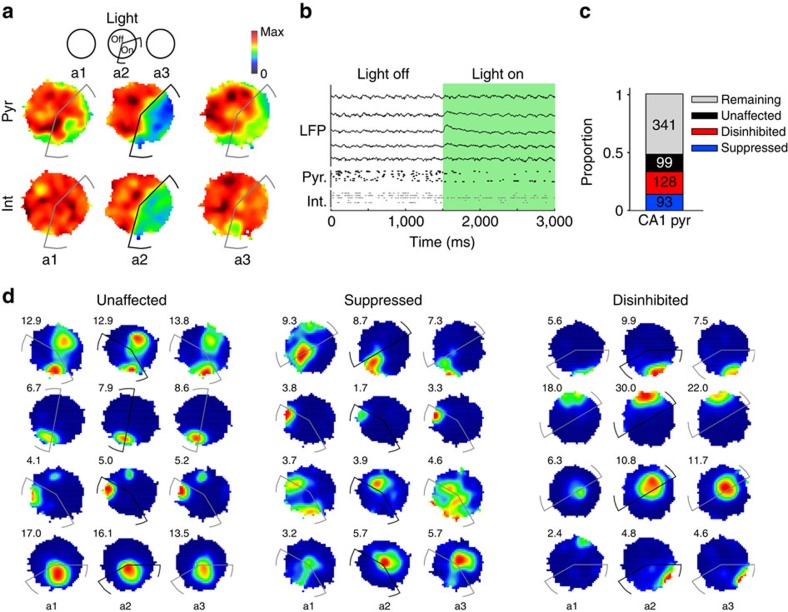
Optogenetic manipulation of CA1 activity during exploration of familiar arena. (**a**) Top: schematic illustrating the three subsequent exploration sessions in a familiar environment (a1–a3). In session a2, the laser light was triggered when the animal entered a specific zone of the arena (‘light zone', indicated by black line in a2). Each exploration session lasted 25 min. Sessions were separated by 25 min sleep/rest sessions in a sleep box. Bottom: cumulated pyramidal cell and interneuron firing maps for a representative recording day in which light application suppressed the rate of the majority of cells in the light zone in a2. Maps were normalized to the maximum rate on the map, as indicated on the map scale. (**b**) Wide-band signal and unit activity while the animal enters the light zone. Green area marks light application. (**c**) Proportions and cell numbers for different cell categories. Rate changes in the light zone during session a2 were used to classify cells. Only neurons passing place-cell criteria in sessions a1 and/or a3 were included in the analysis. Total CA1 pyramidal cells in familiar paradigm, *n*=661 in 9 recording days in 4 animals. (**d**) Place fields of example pyramidal cells. Numbers on the top left of the maps represent the peak firing rates on the map (Hz). Peak rate on the maps were plotted in red, whereas parts of the environment with low rate were plotted in blue.

To quantify how the light has affected the changes of place fields of the different groups, we compared the place field of cells across the three exploration sessions of the familiar environment (Fig. 2). In these experiments a total of 457 cells exhibited a well-defined place field in the familiar environment, of which 93 place cells were strongly suppressed in the light zone, 128 were disinhibited and 99 were not affected during the illumination (see Methods and Supplementary Table 1). The latter served as a control group for the other two categories. In all subsequent statistical analyses, the number of cells in the groups is the same, unless otherwise noted. The remaining 137 place cells that did not exhibit consistent light responses and an additional 204 pyramidal cells that did not show well-defined place fields (using coherence^38^ and sparcity^39^ measures) were not included in the further analysis (see ‘Unit classification by light responses' in Methods).

**Figure 3:**
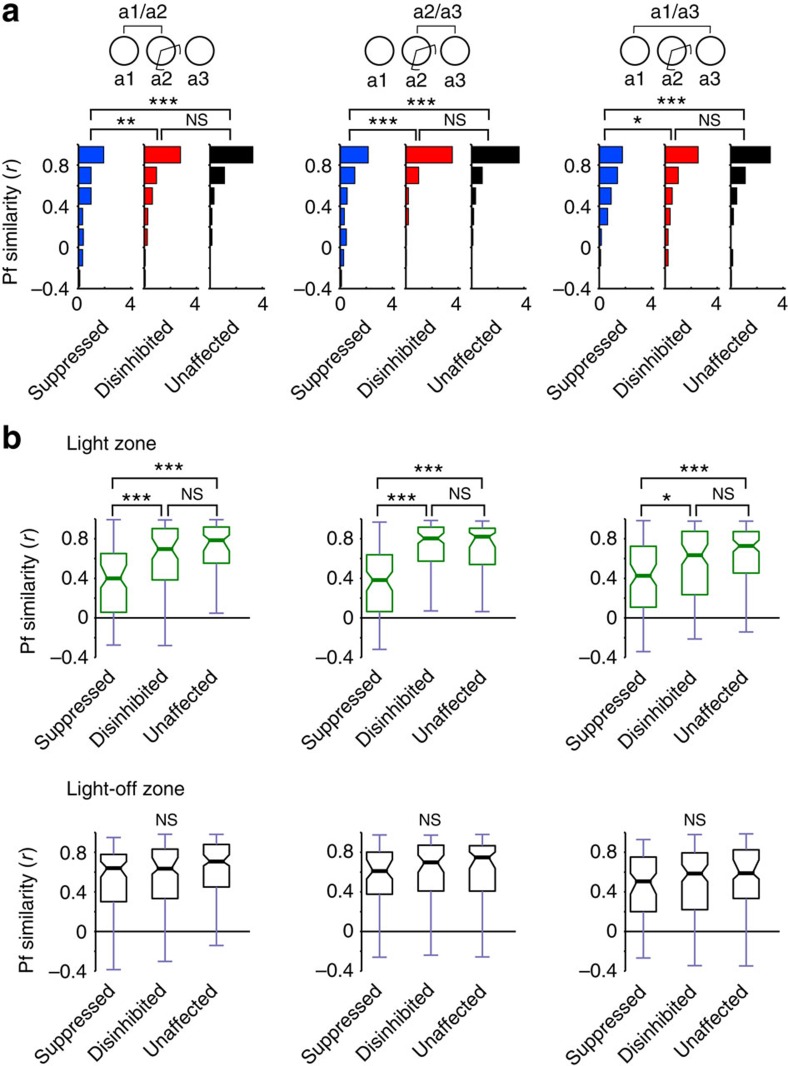
Suppression of CA1 place cell activity leads to sustained reorganization of place fields specifically in the light zone (familiar paradigm). (**a**) Distribution of PFS between exploration sessions for different light-response categories using the entire arena. Suppressed, disinhibited, unaffected Kolmogorov–Smirnov test, a1/a2, suppressed–disinhibited: *D*=0.2605, **P_B2|3_=0.00204; suppressed–unaffected: *D*=0.3897, ***P_B1|3_=0.0000016; disinhibited–unaffected: *D*=0.1598, *P*_B3|3_=0.1042. a2/a3: suppressed–disinhibited: *D*=0.3140, ***P_B1|3_=0.00010; suppressed–unaffected: *D*=0.3187, ***P_B2|3_=0.00024; disinhibited–unaffected: *D*=0.0698, *P*_B3|3_=0.9404. a1/a3: suppressed–disinhibited: *D*=0.2078, *P_B2|3_=0.0321; suppressed–unaffected: *D*=0.3079, ***P_B1|3_=0.00047; disinhibited–unaffected: *D*=0.1556, *P*_B3|3_=0.1213. (**b**) Boxplots showing PFS separately for light zone (top) and outside (bottom). Plots are arranged as in **a**. Boxes extend from lower to upper quartile values of the data, thick line and notch indicate median with 95% confidence interval. Whiskers show the range of the data. Top: Kruskal–Wallis test comparing PFS for cell categories between sessions: a1/a2: *H*=37.98, ***P<0.0000001, *post hoc* Mann–Whitney test: suppressed–disinhibited: *U*=3741.5, ***P_B2|3_=0.000005; suppressed–unaffected: *U*=2312.5, ***P_B1|3_<0.0000001; disinhibited–unaffected: *U*=5661.5, *P*_B3|3_=0.1700. a2/a3: *H*=65.33, ***P<0.0000001, *post hoc* Mann–Whitney test: suppressed–disinhibited: *U*=2461.5, ***P_B2|3_<0.0000001; suppressed–unaffected: *U*=2026.5, ***P_B1|3_<0.0000001; disinhibited–unaffected: *U*=6150.5, *P*_B3|3_=0.7062. a1/a3: *H*=17.50, ***P<0.00016, *post hoc* Mann–Whitney test: suppressed–disinhibited: *U*=4714.5, *P_B2|3_=0.0168; suppressed–unaffected: *U*=2963.5, ***P_B1|3_=0.00006; disinhibited–unaffected: *U*=5557.5, *P*_B3|3_=0.1128. Bottom: Kruskal–Wallis test: a1/a2: *H*=5.14, *P*=0.0765; a2/a3: *H*=3.47, *P*=0.1763; a1/a3: *H*=3.80, *P*=0.1493. *P<0.05, **P<0.01, ***P<0.001, NS, not significant.

First, we checked whether light application initiated changes in the place fields of the three categories of cells during the light-on session itself. To quantify the changes of the place fields, the place rate maps of the groups were compared between the first (light-off) and the second (light-on) exploration sessions of the familiar environment. The place field similarity (PFS) of the maps was measured by the correlation coefficients between the maps. Overall, both the unaffected and disinhibited group showed only small changes of PFS as the result of light application, but the suppressed group exhibited reduced PFS. Accordingly, the PFS distribution was significantly different between the suppressed group and the groups of disinhibited or unaffected neurons (all *P*<0.002, Kolmogorov–Smirnov test), whereas there was no significant difference between the disinhibited and unaffected groups (*P*=0.1; Fig. 3a, left panel).

Light application should modify firing within the zone for affected cells but may also lead to remapping of that portion of the place field that lies outside the zone. We differentiated how the light might affect place fields inside and outside of the light zone by comparing the median PFS of the different groups separately for inside and outside the light zone (Fig. 3b, left panel). Significant differences were seen between the groups only within the light zone (*P*<0.0000001, Kruskal–Wallis test) and only between the suppressed and stable, as well as the suppressed and disinhibited cell groups (all *P*<0.000003, *post hoc* Mann–Whitney test). Therefore, on the population level, light application did not change the overall place fields of cells outside the light zone and within it only the suppressed group was affected. Nevertheless, the alterations of firing rates during exploration might lead to plastic changes that are reflected in the reorganization of place fields in a subsequent visit to the same environment.

To see whether the light-induced activity modification of place cells led to the subsequent remapping of place fields, we then compared PFS in the exploration sessions before and after the light-on session. We found that the light application gave rise to remapping specifically among the suppressed subset of cells. First, the distribution of PFS was significantly different for the suppressed group as compared with the stable and disinhibited groups of neurons (all *P*<0.02, Kolmogorov–Smirnov test) but not between the disinhibited and stable groups (*P*=0.12; Fig. 3a, right panel). Furthermore, the median PFS of the different groups exhibited significant differences only inside the light zone (*P*<0.0002, Kruskal–Wallis test) and only between the suppressed versus stable and suppressed versus disinhibited cell groups (all *P*<0.01, *post hoc* Mann–Whitney test; Fig. 3b, right panel). The place field stability of the unaffected and disinhibited cells was confirmed further when comparing the light-on session with the subsequent exposure to the same environment. The PFS was significantly different only for suppressed group with the other groups (all *P*<0.00005, Kolmogorov–Smirnov test Fig. 3a, centre panel) and these differences were seen only in the light zone (all *P*<0.0000001, *post hoc* Mann–Whitney test; Fig. 3b, centre panel). As the results of all animals were analysed together, it is possible that remapping of place fields of the suppressed cells show significant effect because of the results of a single animal. Therefore, we repeated the analysis by leaving out an animal from the data set and performed the same analysis on the remaining animals. After repeating the analysis by leaving out each animal in turn, we took the least significant (that is, highest) *P*-values from these calculations. Even in this case, the place field of suppressed and unaffected cells was significantly different inside the light zone (highest *P*<0.004). Therefore, after the light was turned off, the place fields of the suppressed group remapped further, leading to a new configuration than that seen in the previous exploration sessions.

**Figure 4:**
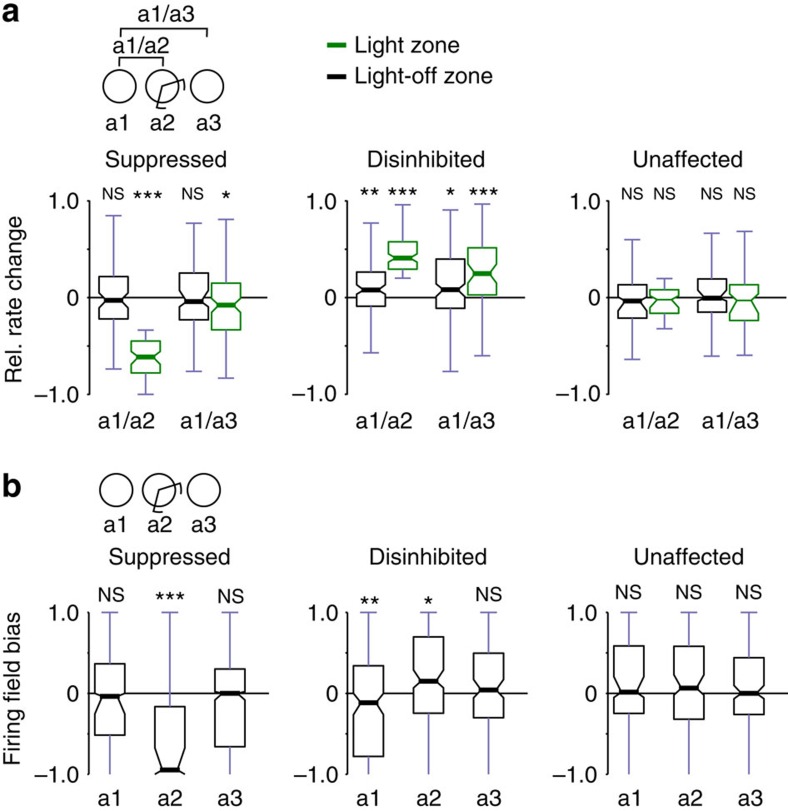
Disinhibition of CA1 place cell activity leads to persistent increase in firing rate. (**a**) Relative firing rate changes between exploration sessions for light zone (green) and outside (black). Binomial test, separately for light zone (ON) and outside (OFF). Suppressed cells: a1/a2, OFF: *P*=0.8358, ON: ***P<0.0000001; a1/a3, OFF: *P*=0.4069, ON: *P=0.0124; disinhibited cells: a1/a2, OFF: **P=0.0019, ON: ***P<0.0000001; a1/a3, OFF: *P=0.0167, ON: ***P<0.0000001; unaffected cells: a1/a2, OFF: *P*=0.1591, ON: *P*=0.4215; a1/a3, OFF: *P*=1.0000, ON: *P*=0.3149. It is noteworthy that a1/a3 ON responses of suppressed cells and the a1/a2 and a1/a3 OFF responses of disinhibitory cells were no longer significant after the leave-out analysis (max *P*s: 0.1249, 0.2451 and 0.6989, respectively). (**b**) Firing field bias for individual exploration sessions. All place map bins with firing rates >1 Hz were included in the firing fields. For a given session, a negative bias indicates a smaller firing field inside than outside the light zone. Binomial test, suppressed cells: a1: *P*=0.1654, a2: ***P<0.0000001, a3: *P*=0.6646; disinhibited cells: a1: *P=0.0232, a2: **P=0.0075, a3: *P*=0.4779; unaffected cells: a1: *P*=0.6817, a2: *P*=0.2615, a3: *P*=1.0000. *P<0.05, **P<0.01, ***P<0.001, NS, not significant.

Overall, the PFS analysis showed that light application only led to specific changes of the place fields in the suppressed group and only within the light zone. However, it is possible that some cells may have altered their firing rate without changing their place fields (rate remapping) as a result of light application^5^. Therefore, we examined how the firing rates of place cells changed across different exploration sessions using a rate remapping score, which measures the difference of firing rates across sessions normalized by the sum of the rates^5^,^22^ (Fig. 4a). As expected, the suppressed cell group reduced their firing inside the light zone and exhibited a rate remapping score significantly lower than zero (*P*<0.0000001, binomial test), when the light-on session was compared with the first familiar exploration session. Disinhibited cells, on the other hand, increased their firing showing a positive rate remapping score (*P*<0.0000001, binomial test; Fig. 4a). To see whether the rate changes during light application were sustained, we compared the rates from the familiar exploration sessions before the light application with the one after. Although the unaffected group did not show sustained differences inside the light zone after the light application (*P*=0.31 binomial test), disinhibited cells showed a significantly positive rate remapping score (*P*<0.0000001 binomial test). When we left out any of the possible animals and repeated the test, similar results were obtained (light-off versus light-on session comparison: disinhibited cells and suppressed cells highest *P*<0.0000001; light-off sessions comparisons: disinhibited cells *P*<0.0001). These results showed that light application resulted in lasting alteration of the firing rates of the affected cells and, specifically, disinhibited cells exhibited rate remapping.

As rate remapping score may be related to PFS, we tested whether these scores correlate. First, we tested whether rate remapping score between the first familiar exploration and the light-on sessions correlated with the lasting place field changes of cells before or after the light application. Only disinhibited cells exhibited stronger correlations when rates were compared inside the light zone (see Supplementary Fig. 4a). Next, we also checked whether rate remapping across the light-off familiar exploration sessions correlated with lasting place field changes, which again resulted in stronger correlations for disinhibited cells inside the light zone (see Supplementary Fig. 4b). However, no significat correlations were seen when rates were compared outside the light zone (see Supplementary Fig. 4c,d). These suggest that strongly disinhibited cells that also exhibited strong rate changes after the cessation of light application also showed stronger PFS changes than weakly rate-changing cells.

Finally, we examined whether the light-induced modification of place cell activity induced any bias in the size of the place fields. For example, the suppressed cell group may show a reduction of firing field size in the light zone not only during illumination but in subsequent sessions as well. (Fig. 4b, see Methods for firing field detection). We compared the firing field sizes of cells in the light zone and outside of it, and calculated a firing field bias by dividing the difference of firing field sizes by their sum (see Methods). We found that the firing fields were reduced for the suppressed group, whereas they were increased for the disinhibited group during the light-on session (all *P*<0.008, binomial test). However, this bias in firing field size did not persist in the subsequent exploration session. When the analysis was repeated by leaving out any of the possible animals, only suppressed group exhibited a significant bias (highest *P*<0.0007). also In addition, it is noteworthy that although disinhibited cells showed a significant negative bias before light application (*P*<0.024), this effect was no longer significant in the leave-out analysis (highest *P*=0.276).

### Unbiased place representation in a novel environment

**Figure 5:**
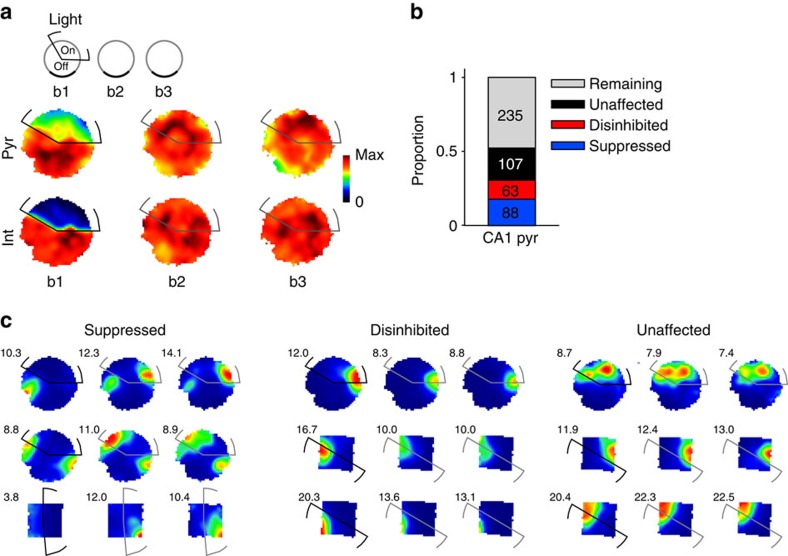
Manipulation of CA1 activity in novel environment. (**a**) Top: simplified schematic showing the three exposures to the novel environment (b1–b3) during the novel experimental paradigm. The laser light was triggered when the animal entered a specific part of the arena (‘light zone'; b1). Sessions lasted 25 min separated by 25 min sleep/rest in a sleep box. Bottom: cumulated firing maps for pyramidal cells and interneurons in a representative recording session. (**b**) Proportions and cell numbers for different cell categories. Only cells passing place cell criteria in sessions b2 and/or b3 were included in the analysis. Total CA1 pyramidal cells in novel paradigm, *n*=493 in 9 recording days in 4 animals. (**c**) Firing maps of example place cells. Numbers on the top left note the top firing rate of the cells on the map (Hz).

**Figure 6:**
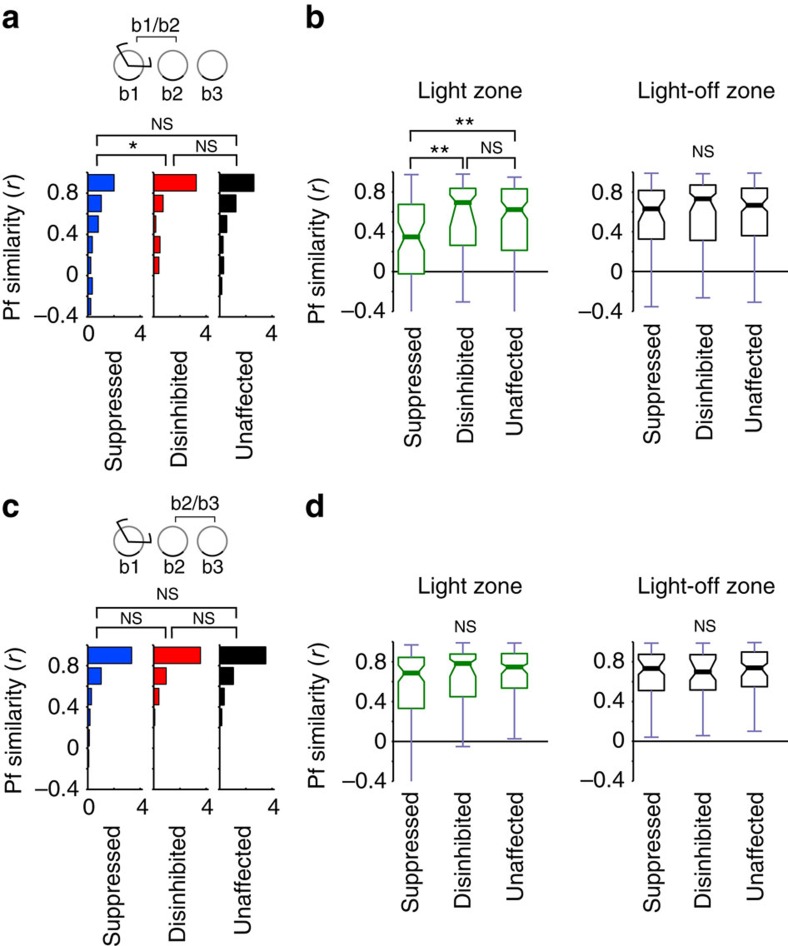
PFSs of CA1 place cells between exploration sessions (novel paradigm). (**a**) Distribution of PFSs between the light-on session and the following exploration session using the entire arena. Kolmogorov–Smirnov test: suppressed–disinhibited: *D*=0.2760, *P_B1|3_=0.0173; suppressed–unaffected: *D*=0.1936, *P*_B2|3_=0.0929; disinhibited–unaffected: *D*=0.1461, *P*_B3|3_=0.3396. (**b**) Boxplots showing PFSs for comparison in **a** separately for light zone (green) and outside (black). Boxes extend from lower to upper quartile values of the data, thick line and notch indicate median with 95% confidence interval. Whiskers show the range of the data. Left: Kruskal–Wallis test: *H*=13.56, **P=0.0011, *post hoc* Mann–Whitney test: suppressed–disinhibited: *U*=1916.0, **P_B1|3_=0.0037; suppressed–unaffected: *U*=3490.5, **P_B2|3_<0.0038; disinhibited–unaffeted: *U*=3224.0, *P*_B3|3_=0.6376. Right: Kruskal–Wallis test: *H*=1.87, *P*=0.3934. (**c**) Comparison between b2 and b3 using entire arena. Kolmogorov–Smirnov test: suppressed–disinhibited: *D*=0.1436, *P*_B2|3_=0.8139; suppressed–unaffected: *D*=1786, *P*_B1|3_=0.2438; disinhibited–unaffected: *D*=0.1078, *P*_B3|3_=0.77213. (**d**) Same as in **b** but comparing sessions b2 and b3. Left: Kruskal–Wallis test: *H*=3.24, *P*=0.1979. Right: Kruskal–Wallis test: *H*=0.36, *P*=0.8351. *P<0.05, **P<0.01, ***P<0.001. NS, not significant.

Next we examined how place cells formed place fields in a novel environment when their firing was disrupted in part of the environment (Fig. 5a and Supplementary Fig. 1b). In this set of experiments, we first recorded exploration in a familiar environment, followed by three exposures to the same previously unseen novel environment. In the first exposure to the novel environment, light was applied in a light zone, as in the previous experiment. The cell groups were established using the light responses during the final laser pulse session recorded at the end of the experimental day (see Fig. 1b–d and Methods). All cell groups formed new place fields in the novel environment; we recorded 88 suppressed, 63 disinhibited and 107 unaffected cells, while a remaining 235 cells did now show reliable place fields (see Methods and Fig. 5b). As expected, the suppressed cell group formed fewer place fields inside the light zone than outside during the light-on session (Fig. 5c). However, they often formed new place fields or expanded their place field into the light zone in the subsequent light-off sessions. The disinhibited and unaffected cells showed similar place fields in the light-on and the subsequent light-off sessions. To quantify how light application might have biased the place field formation of cells in a novel environment, we compared their place maps between the light-on and the subsequent light-off sessions. The distribution of PFSs between the light-on session and the following exploration session exhibited significant differences between the suppressed versus unaffected and suppressed versus disinhibited cell groups (all *P*<0.05, Kolmogorov–Smirnov test; Fig. 6a).

Given that place fields could be readily formed outside the light zone even during the light-on session, we differentially analysed the PFS inside and outside the light zone. The PFS of all the three cell groups was similar across the three explorations of the novel environment outside the light zone (Fig. 6b). However, the PFS of the suppressed cell group was significantly different from that of the unaffected and disinhibited groups in the light zone when the light-on session was compared with the subsequent light-off session in the same environment (all *P*<0.002, Kruskal–Wallis test). Moreover, significant results were obtained between the suppressed and unaffected groups when we left out any of the possible animals (highest *P*=0.0442) but not between the suppressed and disinhibited groups (highest *P*=0.0598). This shows that all cells exhibited similar stable place fields outside the light zone but inside the zone they formed new fields spontaneously once the light was turned off in the following session. Finally, we also examined whether the place maps underwent further changes in the second light-off session. No significant differences were seen between any of the cell groups when the PFS distributions were compared or the median of the groups were compared inside and outside the light zone (Fig. 6c,d).

**Figure 7:**
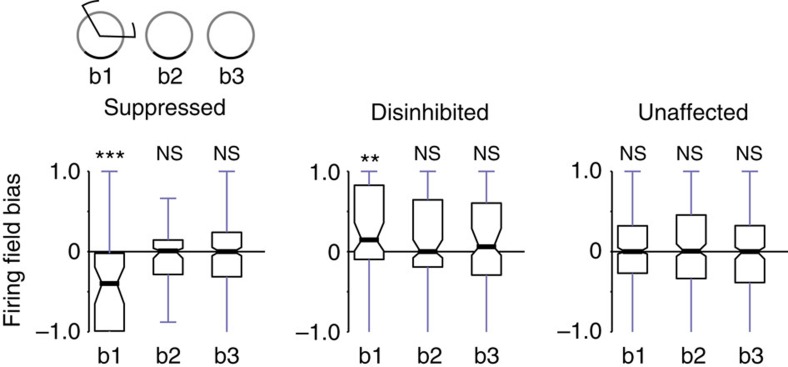
Firing field bias for individual exploration sessions in novel environment. Boxplots of firing rate bias for each group of neurons is shown. All place map bins with firing rates >1 Hz were included in the firing fields. A negative bias indicates smaller firing field inside as compared with outside the light zone in the same session. Binomial test, suppressed cells: b1: ***P<0.0000001, b2: *P*=0.7665, b3: *P*=1.0000; disinhibited cells: b1: **P=0.0086, b2: *P*=1.0000, b3: *P*=0.5258; unaffected cells: b1: *P*=0.8392, b2: *P*=0.4926, b3: *P*=0.8439. *P<0.05, **P<0.01, ***P<0.001. NS, not significant.

Finally, we examined whether any of the cell groups exhibited biased place representations in the light zone during the light-on and in the subsequent light-off sessions. It is possible that these cell groups exhibited a disproportionate place field density in the light zone not only during the light-on session but afterwards as well. We compared the relative firing field density in the light zone and outside (Fig. 7). During the light-on session, as expected, the suppressed cells showed larger firing field density outside the light zone as indicated by a negative firing field bias (*P*<0.0000001, binomial test), whereas the disinhibited cells exhibited a significant positive bias (*P*<0.01), indicating that during illumination they overrepresented the light zone. By leaving out any of the possible animals, suppressed cells remained to show significant bias (highest *P*<0.0000001) but not the disinhibited cells (highest *P*=0.0789). However, firing field density was similar in the second and third exploration of the novel environment for all cell groups when areas inside and outside the light zone were compared. Thus, the group of suppressed cells did not show any bias in the representation of the light zone after release from optogenetic inhibition. Similarly, the disinhibited cells did not over-represent the light zone in the subsequent, unperturbed exploration sessions despite their augmented activity during illumination. This suggests that cells can form multiple place fields in different parts of environments independently from each other and also independently from other CA1 place cells.

## Discussion

Here we showed that suppressing the place-related firing of CA1 cells led to the remapping of their place fields after the light-induced inhibition, triggering lasting changes of hippocampal place maps. By contrast, increasing the firing rate of place cells through disinhibition did not lead to place field remapping; however, cells fired more intensely inside their place fields after the light application than before, albeit at a lower rate than during the disinhibition. These findings demonstrate that place maps are labile when they are expressed and they can be manipulated by biasing the activity of place cells.

Activity-dependent plastic changes have been shown to shape place fields and their stability. Data from multiple experiments suggest that NMDAR-associated, activity-dependent plasticity is required for the stabilization of new place fields^7^,^27^. In our experiments, by applying light in a specific part of the environment, we disrupted the place-related firing of those place cells that had a place field in the light zone. Therefore, the observed remapping of place cells is expected to be caused by activity-dependent plastic changes^40^,^41^, which may have involved long-term depression-like mechanisms^42^ causing the reductions of synaptic weights of the place cells with their place-selective inputs. Indeed, our manipulations prevented the coincident firing of place cells with their presynaptic inputs. Halorhodopsin was localized on both the soma and the dendrites of pyramidal cells and, therefore, it is expected to suppress backpropagating somatic action potentials, reducing dendritic spiking and Ca^++^ levels. Indeed, dendritic spikes that regulate local Ca^++^ levels potentially control plasticity on CA1 pyramidal dendrites^40^,^43^,^44^,^45^.

However, once cells were released from the light-induced suppression of their activity, they rapidly formed new place fields, suggesting that they strengthened their connections with a new set of spatial inputs. This implies that there is an element of randomness how place cells form connections with spatial inputs during remapping, albeit behavioural factors can bias input selection^3^,^8^. Our findings are in agreement with the observation that place cells may form entirely new place fields during the second exposure of a novel environment if the cells were not active long enough within their place field during the first exposure^7^. Thus, place cells may have to be active so that their connections with the spatial inputs can be potentiated and activity is required for the maintenance of these connections as well. If the initial place cell activity is insufficient, or if the maintenance cycle is interrupted, the neurons may establish connections with a new set of spatial inputs leading to remapping. CA1 place cells in a familiar environment receive two dominant spatial inputs: one from the MEC and another one from the CA3 region. Although sensory and path integration input is expected to be directly fed from the MEC^17^,^20^, CA3 may help to establish the correct spatial firing when sensory input from the MEC is incomplete^46^. As many of our suppressed cells formed entirely new place fields, our manipulation may have directly altered MEC-CA1 connection weights. However, considering that pattern completion in CA3 can hinder the expression of new place fields in CA1, the weight of Schaffer collateral synapses may have been altered as well^24^,^46^.

One might expect that the increased firing of cells in specific places triggers the opposite mechanisms: the coincident activation of place-selective inputs with the firing of the cells may lead to potentiation of synaptic weights. This, in turn, can enable the formation of new place fields in the area where excitability of cells is increased through disinhibition. Indeed, it has been suggested that reduction of inhibition can actively shape hippocampal place fields^47^. Surprisingly, this was not the case for our disinhibited cells. They increased their overall firing inside their place fields; however, new place fields were rarely formed. This is in agreement with previous observations that silencing of CA1 interneurons does not alter the place fields of cells^48^. Moreover, disinhibition may not have directly facilitated the emergence of dendritic plateau potentials in relation to a new set of spatial inputs^26^. Interestingly, our disinhibited cells fired more intensely inside their place fields even after the light-induced disinhibition ceased, possibly by activity-dependent potentiation mechanisms^40^,^49^. Although it is expected that place cells express place-specific subthreshold depolarization outside their place fields^10^, the increased excitability of place cells through disinhibition may not have been sufficient to reach the action potential threshold in familiar environments. Moreover, it is also possible that the pattern completion role of CA3 in familiar environments may prevent the expression of new place fields if the old place fields were still active^22^,^23^. Therefore, place cells may need to weaken their existing spatial inputs before new fields can be formed.

As in the familiar environment, even in the novel environments disinhibition had little influence to bias the formation of new place fields. Minimal remapping has been seen between the light-on session and in the subsequent sessions in the same environment for disinhibited cells. Although the firing rate of cells was higher in the light-on session than later, no bias has been seen in terms of place field coverage for the disinhibited cells in the light zone after the light was turned off. Hence, the formation of new place fields may be rapidly determined by the combination of spatial inputs that preferably drive that cell. For the cells that were suppressed by light, as expected, the light zone was under-represented in terms of place-field coverage when the light was initially applied. However, place cells rapidly formed new place fields when the light was turned off and they represented equally well areas inside and outside the light zone. Importantly, these cells exhibited minimal remapping of their place fields outside the light zone. Therefore, the emergence of new place fields in the light zone did not bias their existing place fields outside. A possible explanation for this finding is that once a novel environment is presented to the animal, the combination of spatial inputs enabled the suppressed cells to form associations both inside and outside the light zone. Indeed, synaptic changes on the inhibited cells may have already occurred during the light-on session, considering that somatic action potentials are not required for plasticity in CA1 cells^44^. Alternatively, inhibited cells may have formed new associations with spatial inputs in the light zone only when they were released from the light-induced inhibition.

Both the experimental results of the novel and familiar paradigms illustrate the relative independence of CA1 cells to form place fields, that is, the remapping of the suppressed cells had little influence on the spatial coding of other cells. Surprisingly, the light-induced inhibition of interneurons had also little effect on the spatial firing of unaffected group even though the activity of 80% of our recorded interneurons was suppressed by the light. Therefore, local inhibitory-feedback mechanisms had little effect on the spatial tuning of place cells; their place-related activity is largely driven feedforwardly from MEC and CA3 inputs. In addition, the same place cell may form independently new place fields in spatially distinct parts of an environment as illustrated in the novel environment, where suppressed place cells expressed novel place fields in the light zone without altering already established place fields outside the light zone.

What might be the mechanism underlying place field remapping? Our results point to essential steps that are needed when previously stable place fields remap in a familiar environment. Critically, this process would involve the weakening of connections that promoted the expression of the previous place map. The reduction of connection weights with the old synaptic inputs may be a gradual process. This may explain why old maps and new maps initially flicker when new place fields are formed as results of goal learning^50^ or why place maps of similar recording enclosures converge to each other slowly^51^,^52^. Inhibitory mechanism causing the desynchronization of spatial inputs with the cell's activity may be the first step of such a process. Some degree of destabilization of existing synaptic weights may be needed for the remapping of place fields even in a novel environment, to ensure that novel assemblies are activated, that is, that place cells fire at different relative spatial displacement from each other in different environments. Therefore, destabilization of maps may be the first step of any remapping process. Indeed, it was observed that inhibitory neurons fire initially stronger than later when animals are placed in a novel environment^6^.

Our results also demonstrated that disinhibition might be a way how rate remapping may occur. We observed that transient increase in the excitability of cells within their place fields enable these cells to undergo rate remapping by upregulating their firing without changing their place fields. Therefore, even a place-independent reduction of inhibition on a cell can lead to place-field-specific increases of its spatial firing. Accordingly, our results suggest that rate remapping does not require place-field-specific control; location-independent modulation of the excitability of a place cell is sufficient to cause it.

## Methods

### Surgery

Five male adult rats (Long Evans, 300–500 g) were injected with a recombinant AAV to express Halorhodopsin-YFP in the dorsal CA1 area (AAV2/1.CaMKIIα::eNpHR3.0-YFP^53^, obtained from the Penn Vector Core facility, 1.6 × 10exp13 genome copies per ml; Addgene 26971). The virus delivery protocol was optimized in a total of 14 animals to ensure high and homogeneous expression in the dorsal CA1 area. The final set of coordinates with only minor changes was used in a total of nine animals, which includes the animals implanted with microdrives. Virus was injected at four sites into dorsal CA1 of the right hemisphere: site at the following anterior-posterior (AP), mediolateral (ML) and dorsoventral (DV) coordinates 1: −3.0 AP, 2.2 ML, 2.1 DV; site 2: −3.7 AP, 2.9 ML, 2.0 DV; site 3: −4.3 AP, 3.5 ML, 2.0 DV; and site 4: −5.0 AP, 4.2 ML, 2.2 DV. Before injection, the virus solution was diluted 1:13 with physiological NaCl solution. The virus was loaded into a calibrated glass capillary with a tip pulled to an inner diameter of ∼15 μm. At each injection site, after capillary insertion and a wait period of 3 min, 300 nl diluted virus solution was pressure injected over 4 min. Three and a half weeks after virus injection, animals were implanted with 15 independently movable wire tetrodes under deep anaesthesia using isoflurane (0.5–2%), oxygen (1–2 l min^−1^) and an initial dose of buprenorphine (0.1 mg kg^−1^). Tetrodes were attached to the 15-tetrode microdrive assemblies, enabling their independent movement. The tetrodes were constructed from four individual tungsten wires, 12 μm in diameter (H-Formvar insulation with Butyral bond coat, California Fine Wire, Grover Beach, CA), twisted and then heated, to bind them into a single bundle. The tips were then gold plated to reduce their impedance to 200–300 kΩ.

A 200 μm per 0.48 NA optic fibre stub (Doric Lenses) located in the centre of the tetrode array was used to apply laser light directly to the dorsal CA1 area. The light transmission efficiency of each fibre stub was measured before implantation. During surgery, a craniotomy was prepared above the dorsal hippocampus centred at AP=−4.0; ML=3.0. Two stainless steel screws inserted through the skull above the cerebellum served as ground and reference electrodes, and six additional screws were used to permanently attach the microdrive assembly to the skull. Implantation was performed such as to position the tip of the optic fibre at a depth of 1.7 mm. The paraffin wax-coated electrodes and microdrives were then daubed with bone cement to encase the electrode-microdrive assembly and anchor it to the screws in the scull. Following a recovery period of 7 days, the tetrodes were lowered to their target locations over a further period of around 7 days. Tetrode locations were identified by electrophysiological markers such as theta band power, sharp wave polarity and the presence of ripple oscillations, and by extrapolating location of the electrodes by tracing the distances back along each electrode tract according to the daily advancement of the recorded electrodes. Implanted animals were housed individually in a separate room under a 12 h light/12 h dark cycle with *ad libitum* access to water and they were maintained in a food-deprived state between 85 and 90% (plus an incremental 5 g per week) of their postoperative weight. Experiments were performed during the light phase. All rats used in this study were naive and not used for additional procedures before surgery.

All procedures involving experimental animals were carried out in accordance with Austrian (Austrian federal law for experiments with live animals) animal law under a project license approved by the Austrian Federal Science Ministry.

### Data acquisition

Thirty-two-channel unity-gain preamplifier panels (Axona Ltd, St Albans, Hertfordshire, UK) were used to reduce cable movement artefacts. Wide-band (0.1/1 Hz–5 kHz) recordings were taken and the amplified local field potential and multiple-unit activity were continuously digitized at 24 kHz using a 64-channel data acquisition system (Axona Ltd). Two red LEDs mounted on the preamplifier headstage were used to track the location of the animal.

Green/yellow laser light for NpHR activation was provided by a 561-nm diode-pumped solid-state laser (DPSS) laser system equipped with an acousto-optic modulator (Omicron). The light was coupled into an optic fibre connected to a fibre-optic rotary joint (Doric Lenses) from where a 200 μm per 0.48 NA patch cord transmitted the light to the microdrive. Laser intensity was set to reach 25 mW total power at the tip of the implanted fibre stub. Data were recorded 6–7 weeks after AAV injection, to ensure sufficient NpHR-YFP expression levels.

### Behavioural paradigms

Data were recorded while the animals explored different arenas or rested in a sleep box (see Supplementary Fig. 1). The sleep box was small (20 cm × 27 cm) with 60 cm high walls and cushioned with a terry towel for the animal to sleep/rest comfortably. During training and electrode positioning, the animals were familiarized with a 120-cm circular environment with 20 cm high walls (minimum of 60 min of exposure per day for at least 7 days) that served as the familiar arena in all experiments. Curtains were used to enclose this arena and provide a stable set of external cues. In all exploration sessions, small food pellets were dropped at random from an automated overhead system (2–3 min^−1^), to motivate the animals to explore the entire arena. For recordings in a novel environment, several other arenas with different sizes, shapes and textures were used. In addition, curtains were opened to provide novel distal room cues.

Typical recording days consisted of ten sessions: four exploration sessions flanked by five sleep sessions and a final test session where brief laser pulses were applied while the animal still rested in the sleep box. Typically, sleep and exploration sessions lasted 25 min, whereas the laser test session lasted 18 min.

Data were recorded in two different behavioural paradigms. In the ‘Familiar paradigm' (Supplementary Fig. 1a), the animals first explored the familiar arena. After visiting a different arena, the familiar arena was explored again, but laser illumination was automatically triggered when the animal entered a specific part of the arena (we refer that that part of the arena as the ‘light zone' in all exploration sessions, that is, also when no laser illumination was triggered in exploration sessions before or after the illumination session). Finally, the same arena was explored again. The ‘Novel paradigm' (Supplementary Fig. 1b) also started with exploration of the familiar arena. For the subsequent session, the animal was placed in a novel arena and laser application was triggered when the animal entered the light zone. Following this, the animal explored the same arena two more times. In both paradigms, all exploration sessions were flanked by sleep. Triggers for laser illumination were generated by a custom script running on the data acquisition system that continuously analysed the animal's position. The light zone was defined by a centre position and an angle between 120° and 180° such that it covered one-third to half of the arena (see Supplementary Fig. 1). The initial angle defining the illumination zone was random and thus random with respect the hippocampal place fields as well. Every day, a novel illumination zone that had ∼50% overlap with the previous day's zone was defined. During the course of the project and also within individual animals, the angle defining the size of the illumination zone was increased, to include more place fields in the light zone. For the Familiar paradigm, in four sessions the zone was <180° and in five sessions it was equal to 180°. For the Novel paradigm, in six sessions the zone was <180° and in three sessions it was equal to 180°. Illumination in the light zone was interrupted when the animal did not move for >2 s.

Three rats contributed data for both behavioural paradigms. Two rats contributed data for either the Familiar or the Novel paradigm only. No animal was excluded from analysis. No randomization was used to assign recording days to behavioural paradigms. Instead, we typically recorded the Novel paradigm and the Familiar paradigm alternatingly.

After completion of the experiments, the rats were deeply anaesthetized and perfused through the heart first with PBS followed by a 4% buffered formalin phosphate solution for the histological verification of electrode tracks and optic fibre position. Furthermore, NpHR-YFP expression in dorsal CA1 was verified in each animal by checking fluorescence of the yellow fluorescent protein (YFP) tag.

### Spike sorting and unit classification

Unit isolation and clustering procedures have been described before^54^. Briefly, after resampling of the raw data to 20 kHz, action potentials were extracted from the digitally high-pass-filtered (0.8–5 kHz) signal. The power computed in a sliding window (12.8 ms) and action potentials with a power of >5 s.d. from the baseline mean were selected. The spike features were then extracted using principal components analyses. The detected action potentials were then segregated into putative multiple single units using an automatic clustering software^55^ (http://klustakwik.sourceforge.net/). Finally, the generated clusters were manually refined by a graphical cluster cutting programme. Only units with clear refractory periods in their autocorrelation and well-defined cluster boundaries were used for further analysis. Periods of waking spatial exploration, immobility and sleep were clustered together. Stability of the cells was verified by plotting spike features over time. In addition, an isolation distance (based on Mahabalonis distance^55^) was calculated to ensure the spike clusters did not overlap during the course of the recordings. CA1 pyramidal cells and interneurons were discriminated by their autocorrelations, firing rate and wave forms^56^,^57^. In total, we recorded 1,214 CA1 cells (1,154 pyramidal cells and 60 interneurons.

### Theta and sharp-wave detection

To identify periods of theta activity, the theta/delta power ratio was measured in 1,600 ms segments (800 ms steps in between measurement windows), using Thomson's multi-taper method^58^. Exploratory epochs included periods of locomotion or the presence of theta oscillations (as seen in the theta/delta ratio), including a <2.4 s (that is, two consecutive windows) transient from immobility segments. Waking immobility sessions were selected when both the speed and theta–delta ratio were below a pre-set threshold for at least 2.4 s interval. Sleep sessions were recorded separately and were identified by occasional occurrence of rapid eye movement sleep-associated-theta periods and the presence of slow-wave oscillations. For the detection of SWRs, local field potentials were band-pass filtered (150–250 Hz) and a reference signal (from a channel that did not contain ripple oscillations) was subtracted to eliminate common-mode noise (such as muscle artifacts). The power (root mean square) of the filtered signal was calculated for each electrode and summed across electrodes designated as being in the CA1 pyramidal cell layer. The threshold for SWR detection was set to 5 s.d. above the background mean. The SWR detection threshold was always set in the first available sleep session and the same threshold was used for all other sessions.

### Generation and comparison of spatial firing maps

To compute firing rate maps, arenas were divided into 4 cm × 4 cm bins. For each bin, spikes were counted and divided by the animal's occupancy time in that bin. Raw rate maps were smoothed using a two-dimensional Gaussian kernel. To compare place maps between exploration sessions, firing rates of all bins visited in both sessions were correlated bin-by-bin to calculate PFS. Therefore, a high PFS indicates high stability of the place maps between the two sessions. To compare the place representations inside or outside the light zone between two exploration sessions, place maps were generated for these zones separately and smoothing was limited to within a given zone to prevent zone-specific effects from spuriously spreading into the neighbouring zone as a result of smoothing. When the angle defining the light zone was smaller than 180°, we used a proportion of the light-off zone defined by the same angle to avoid biasing the place map comparisons, that is, if the light zone was defined by a 150° angle, a 150° proportion of the light-off zone was used. Cumulated rate maps were constructed by adding up the firing rates of all individual pyramidal cells or interneurons, respectively, for each bin.

### Unit classification by light responses

Sleep: to classify the light response of individual units independently of their spatial firing properties, their firing during application of brief laser pulses was used. Laser pulses (400 ms) were applied at 0.5 Hz for typically 18 min, while the animal rested in the sleep box at the end of the recording day. Neurons in the ‘suppressed' group exhibited a firing rate reduction >33% during illumination (see Fig. 1c), whereas ‘disinhibited' neurons showed an increase of >33%. A Wilcoxon signed-rank test was performed to confirm that the rate changes were statistically significant (*P*<0.05). According to these criteria, the activity of *n*=386 pyramidal cells and *n*=46 interneurons was suppressed by light, and *n*=244 pyramidal cells were disinhibited during illumination. The group of the ‘remaining' cells (*n*=524 pyramidal cells, *n*=14 interneurons) thus comprised all neurons that did not respond to light application or that exhibited random rate fluctuations, therefore not passing the significance test.

Familiar paradigm: we only included CA1 place cells with place-field sparcity <0.3 (ref. 38) and coherence >0.55 (ref. 39), and a minimal firing rate of 0.25 Hz during exploration sessions a1 and/or a3 in the analysis (see Supplementary Fig. 1 for the detailed behavioural paradigm). Cells were classified as ‘suppressed' if their firing rate in the light zone decreased >50% during illumination in exploration session a2 compared with session a1, where firing was unperturbed. Only neurons that in sleep also exhibited a drop-in firing during application of brief laser pulses were used to exclude neurons with random rate fluctuations from the analysis. Neurons with a firing rate increase >50% in session a2 during illumination in the light zone compared with session a1 and a positive light response during laser pulse application during sleep were classified as ‘disinhibited'. ‘Unaffected' neurons exhibit rates within ±50% during illumination in session a2 and also during laser pulses applied in sleep. The ‘remaining' group thus comprised all neurons that did not pass place cell criteria or did not exhibit consistent light-dependent rate modulation during sleep and explorations. According to these classification criteria, 93 place cells were suppressed during exploration, 128 were disinhibited and 99 were not affected by light.

Novel paradigm: as in this paradigm CA1 illumination was done the first time the animals explored a novel arena, light responses could not be determined by comparing firing rates during illumination in the light zone with the rates in previous exploration sessions. We therefore classified the neurons using the light response during rest as described above. Only neurons passing place cell criteria (sparsity <0.3, coherence >0.55, mean firing rate >0.25 Hz) in sessions b2 and/or b3 were used. A total of 258 pyramidal cells passed place cell criteria. Of these, 88 were suppressed, 63 were disinhibited and 107 were not affected by light.

### Firing rate and firing field analysis

To compare firing rates between two sessions, we calculated the relative firing rate change by dividing the signed difference between the mean firing rates by the sum of the mean rates (that is, *c*=(r2−r1)/(r2+r1), where r1 and r2 denote the mean firing rates in the two sessions that are compared^5^,^22^. This score is always between −1 and 1, and the extreme values −1 and 1 mean that a neuron is firing exclusively in one of the two sessions. The fold change r2/r1 can be directly calculated from the relative score using r2/r1=(*c*+1)/(1−*c*).

As a complementary measure for place cell activity that is independent of firing rate, we quantified the size of the firing fields. For this, we used the place maps constructed as described above, and the firing field was defined as the proportion of spatial bins with a firing rate >1 Hz. To check whether a neuron fired preferentially in the light zone or outside of it, we calculated a firing field bias by dividing the difference of the firing fields by the sum of the firing fields. A negative bias therefore indicates that the spatial firing of a neuron is weaker in the light zone than outside and, conversely, a positive bias shows a preference of the neurons to have firing fields outside the light zone. If the angle of the light zone was <180°, an area of the same size from the light-off zone was used for comparisons as described above.

### Immunohistochemistry

Immonostainings for parvalbumin and somatostatin were performed on tissues from two animals, wherein we allowed only 3–4 weeks of expression such that NpHR-YFP expression in pyramidal neurons was still weak. This allowed optimal contrast to visualize NpHR-YFP expression in interneurons. The tissue was sliced to generate 40–80 μm sections. The primary antibody (rabbit polyclonal antibody to PV 1:2,000, Abcam ab11427; rabbit polyclonal to Somatostatin-14 1:500, T-4103, Peninsula Laboratories) was applied overnight in permeabilization buffer (PBS containing 1% BSA, 0.3% Triton X-100). After extensive washing, the secondary antibody (Alexa Fluor 594 goat anti-rabbit IgG, Life Technologies A-11037) was applied for 2 h in 1:3 diluted permeabilization buffer. An anti-green fluorescent protein antibody (fluorescein isothiocyanate-conjugated goat polyclonal anti-green fluorescent protein 1:500, Abcam ab6662) was used to amplify the NpHR-YFP signal. After extensive washing, cultures were mounted on glass slides and stored at 4 °C.

### Statistical analyses

In bar plots, mean+s.e.m. are shown. Boxplots show the median of the data set with a notch, indicating the 95% confidence interval of the median determined by bootstrapping. Boxes extend from lower to upper quartile, whiskers indicate the range of the data. Statistical analyses were done in Python using the scipy package for scientific computing (www.scipy.org). All tests reported were done two-sided. As the data typically was not normally distributed, we used non-parametric tests. The only exception was analysis of behavioural data (Supplementary Fig. 2a), where we used a paired *t*-test. Normal distribution of these data was verified using a Shapiro–Wilk test. *P*-values and statistical tests for all experiments are reported in the appropriate figure legends. We did a Holm's sequential Bonferroni procedure for multiple comparisons when several pair-wise comparisons were done. Accordingly, *P*-values were corrected and specified with the following nomenclature: *P*_B1|3_ indicates the most significant of three pair-wise comparisons, *P*_B2|3_ the second-most significant of three comparisons and so on. No blinding was done for data analysis. Sample sizes are in the range commonly used in the field and they were not determined before the experiments using statistical methods.

### Data availability

The data that support the findings of this study are available from the corresponding author on request.

## Additional information

**How to cite this article:** Schoenenberger, P. *et al*. Activity-dependent plasticity of hippocampal place maps. *Nat. Commun.* 7:11824 doi: 10.1038/ncomms11824 (2016).

## Supplementary Material

Supplementary Information Supplementary Figures 1 - 4 and Supplementary Table 1
