# Supplementary material for: Activity-dependent plasticity of hippocampal place maps
Source: Nat Commun. 2016 Jun 10;7:11824. doi: 10.1038/ncomms11824 (PMC4906387; doi:10.1038/ncomms11824)
Supplement: Supplementary Information — Supplementary Figures 1 - 4 and Supplementary Table 1 [file ncomms11824-s1.pdf]

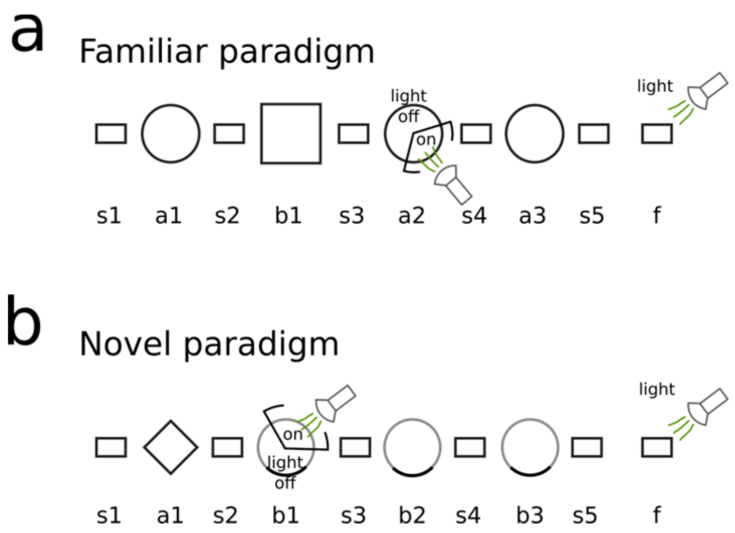

Supplementary Figure 1: Detailed behavioral paradigms.

(a) 'Familiar paradigm'. This paradigm consisted of a series of exploration sessions that were flanked by sleep/rest. Each exploration session lasted for 25 min. First, the animal explored a familiar arena (a1). After exploration of another arena (b1), the animal visited the first arena again, but each time the animal entered the light zone, the laser was triggered to illuminate dorsal CA1 (a2; indicated by the flash light). Subsequent to this, the animal explored the first arena again (a3). Sleep/rest was recorded for 25 min while the animal was in a small sleep box cushioned with a terry towel. At the end of the recording day, responses to brief laser pulses were recorded while the animal stayed in the sleep box (f). (b) The 'Novel paradigm' started with the animal visiting a familiar arena (a1).Then, the animal explored a novel arena and each time the light zone was entered, laser illumination was triggered (b1). Following this, this arena was explored two more times (b2,b3). All sessions were flanked by sleep, both sleep and exploration sessions lasted 25 min. At the end of the recording day, responses to brief laser pulses were recorded while the animal stayed in the sleep box (f).

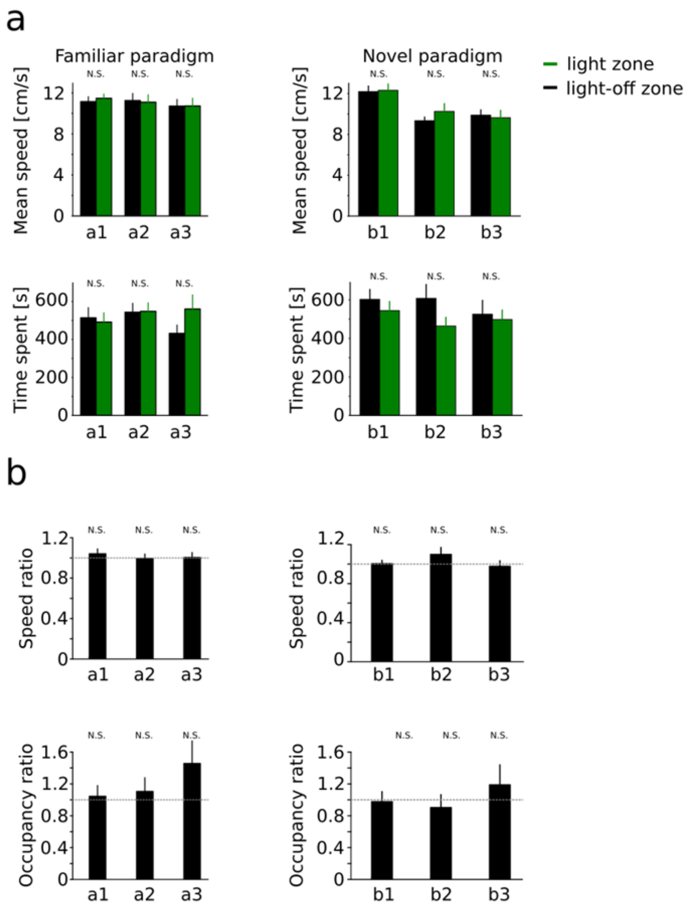

Supplementary Figure 2: Animal behavior was not affected by light application.

(a) Mean running speed of the animal and time spent inside and outside the light zone during exploration. The animal did not prefer or avoid the light zone during illumination. Error bars represent mean + s.e.m. Paired t-test, Familiar paradigm, mean speed (n=9), a1: t=-0.6226, P=0.5509; a2: t=0.3076, P=0.7662; a3: t=-0.0266, P=0.9794. Time spent (n=9),a1: t=0.3089, P=0.7653; a2: t=-0.0625, P=0.9517; a3: t=-1.3139, P=0.2253. Novel paradigm, mean speed (n=9): b1: t=-0.2423, P=0.8146; b2: t=-1.211, P=0.2604; b3:t=0.3741, P=0.7180. Time spent (n=9), b1: t=0.7110, P=0.4973; b2: t=1.4501, P=0.1851;b3: t=0.2752, P=0.7901. N.S., not significant. (b) Ratios of running speed and occupancy inside and outside the light zone. Error bars represent mean + s.e.m. Binomial test: Familiar paradigm, speed ratio, a1: P=1.000 ; a2: P=1.000; a3: P=1.000. Occupancy ratio, a1: P=1.000 ; a2: P=0.508; a3: P=1.000. Novel paradigm, speed ration, P=1.000 ; a2: P=0.508; a3: P=1.000. Occupancy ratio, P=1.000 ; a2: P=0.508; a3: P=1.000.

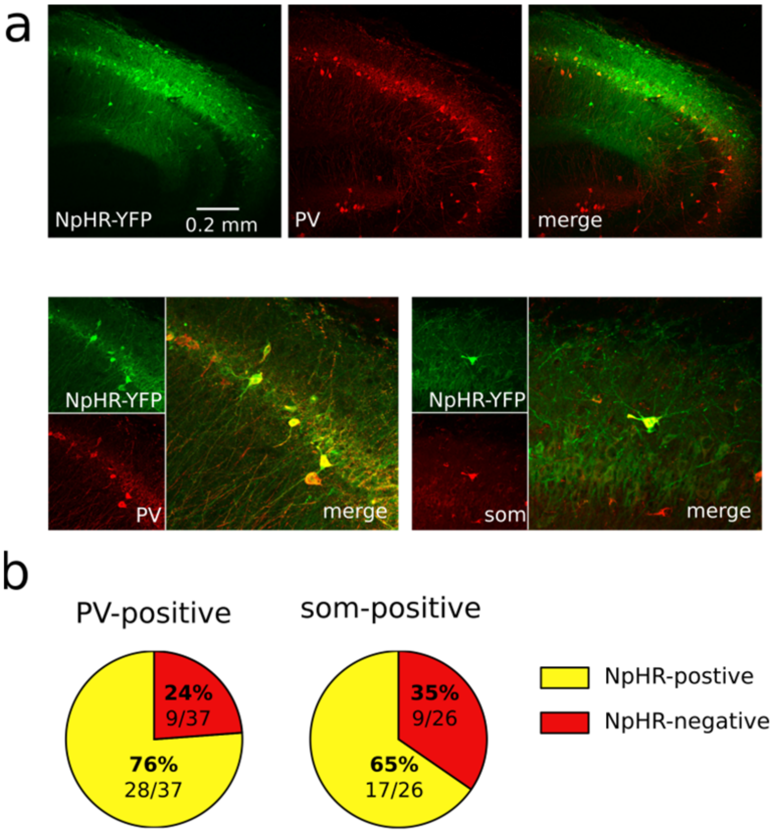

Supplementary Figure 3: NpHR-YFP expression in interneurons.

(a) Top: Dorsal CA1 transduced with NpHR-YFP, co-immunostainings against PV. Bottom: Confocal images showing PV-positive or somatostatin-positive interneurons expressing NpHR-YFP. For optimal visualization of NpHR-YFP expression in interneurons, histology was done 3 weeks after virus injection when expression in pyramidal neurons was still weak. (b) Quantification of NpHR-expression in PV- and somatistatin-positive interneurons.

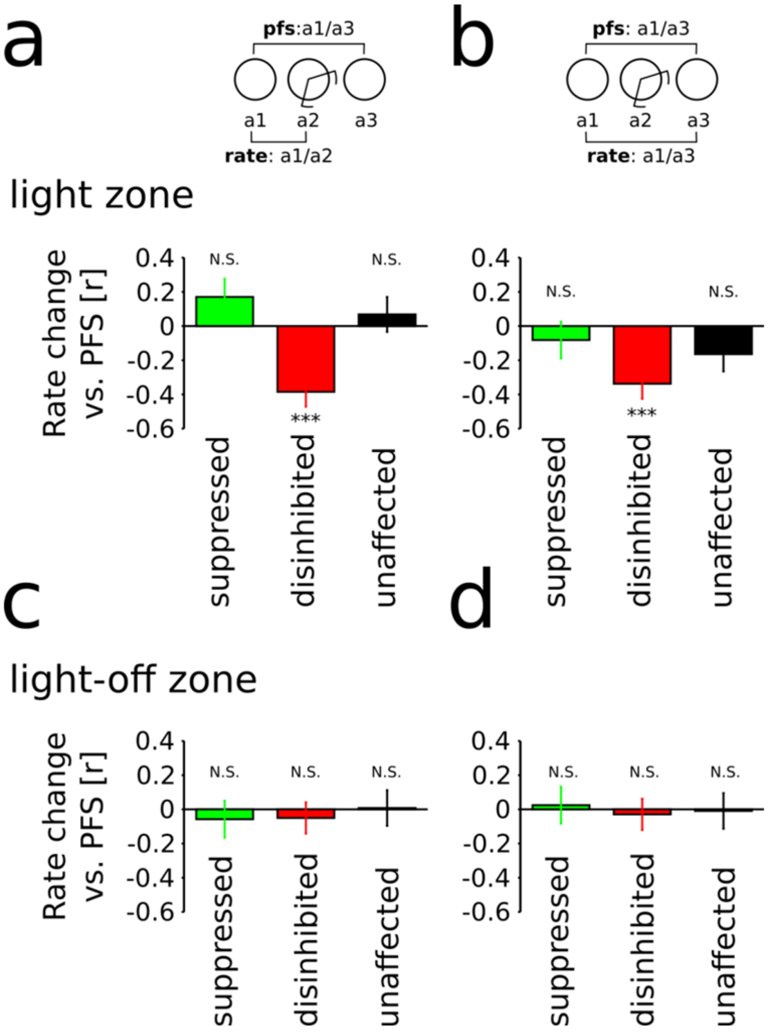

Supplementary Figure 4: Correlation between rate change score and place field similarity for light zone and the light-off zone.

(a,c) Rate change score between sessions a1 and a2 was correlated with PFS between sessions a1 and a3. (b,d) Rate change score between sessions a1 and a3 was correlated with pfs between sessions a1 and a3. p-values for individual correlations: (a) suppr: P=0.1125; disinh: ***P<0.0001; unaff: P=0.5068. (b) suppr: P=0.4490; disinh: ***P=0.0002; unaff: P=0.1083. (c) suppr: P=0.5946; disinh: P=0.5821; unaff: P=0.9390. (d) suppr: P=0.8202; disinh: P=0.7475; unaff: P=0.9299.

Single unit light responses								
	Sleep/rest					Exploration		
**Familiar paradigm**	**Suppressed**	**Activity / s.e.m.**	**Disinhibited**	**Activity / s.e.m.**	**Ratio suppr/disinh**	**Suppressed**	**Disinhibited**	**Ratio suppr/disinh**
Animal 1, day 2	18	24.6 ± 4.9%	10	172.5 ± 6.7%	1,80	8	9	0,89
Animal 1, day 4	49	21.7 ± 2.6%	4	217.8 ± 28.7%	12,25	13	3	4,33
Animal 2, day 2	49	23.7 ± 2.9%	17	231.0 ± 19.1%	5,13	34	11	3,09
Animal 2, day 4	36	27.5 ± 3.4%	14	272.7 ± 39.3%	2,57	16	11	1,45
Animal 3, day 1	0	N/A	3	251.5 ± 43.1%	0,00	1	3	0,33
Animal 3, day 2	11	43.0 ± 4.6%	23	138.3 ± 11.9 %	0,48	5	23	0,22
Animal 4, day 2	10	27.3 ± 5.1%	27	257.8 ± 20.0%	0,37	7	21	0,33
Animal 4, day 4	13	33.1 ± 4.5%	44	266.5 ± 16.0%	0,30	8	38	0,21
Animal 4, day 5	1	38.3 ± 0.0%	14	264.4 ± 14.9 %	0,07	1	9	0,11
**Novel paradigm**								
Animal 1, day 1	28	35.5 ± 4.0%	6	192.6 ± 16.1%	4,67	15	4	3,75
Animal 1, day 3	22	20.1 ± 3.1%	7	181.1 ± 12.0%	3,14	11	5	2,20
Animal 2, day 1	41	23.5 ± 3.2%	8	187.7 ± 18.4%	5,13	15	4	3,75
Animal 2, day 3	38	25.4 ± 3.0%	10	291.1 ± 32.7%	3,80	15	7	2,14
Animal 2, day 5	20	22.6 ± 4.7%	5	203.6 ± 26.4%	4,00	5	2	2,50
Animal 4, day 1	5	49.1 ± 7.7%	5	195.8 ± 20.8%	1,00	4	5	0,80
Animal 4, day 3	11	38.3 ± 5.1%	23	248.1 ± 23.5%	0,48	6	19	0,32
Animal 5, day 1	21	28.4 ± 3.8%	7	171.8 ± 13.5%	3,00	9	5	1,80
Animal 5, day 2	13	34.7 ± 5.1%	17	168.0 ± 10.5%	0,76	8	13	0,62

Supplementary Table 1: Number of cell categories and their light responses in different sessions
